# Metabolomic Investigation on Fermentation Products of *Achyranthes japonica* Nakai by *Lactobacillus plantarum*

**DOI:** 10.4014/jmb.1910.10057

**Published:** 2019-12-09

**Authors:** Chang-Wan Lee, Do Yup Lee

**Affiliations:** 1 Department of Bio and Fermentation Convergence Technology, BK2 PLUS Program, Kookmin University, Seoul 02707, Republic of Korea; 2 Bio R&D Center, SK Bioland Co., Ltd., Ansan 15407, Republic of Korea; 3 Department of Agricultural Biotechnology, Center for Food and Bioconvergence, Research Institute for Agricultural and Life Sciences, Seoul National University, Seoul 08826, Republic of Korea

**Keywords:** *Achyranthes japonica* Nakai, *Lactobacillus plantarum* fermentation, LC-orbitrap MS, GC-TOF MS, metabolic profiling

## Abstract

Fermentation has recently re-emerged as an approach for improved functionality of food products in addition to the traditional roles such as shelf life, taste, and texture. Here, we report dynamic changes in the metabolite profiles of *Achyranthes japonica* Nakai by *Lactobacillus plantarum* fermentation, primarily, the significant increases in representative functional ingredients, 20-hydroxyecdysone and 25S-inokosterone. Additionally, untargeted metabolite profiling showed 58% of metabolites underwent significant alteration. The most dynamic change was observed in cellobiose, which showed a 56-fold increase. Others were sugar alcohols and amino acids, while lyxitol and erythritol that were among the most dynamically down-regulated.

Achyranthis Radix is an herbal medicine derived from *Achyranthes bidentata* Blume and *Achyranthes japonica* Nakai (AJN), which has been recognized for improved sinew and bone health. Recent related scientific evidence has also reported hypoglycemic, anti-inflammatory, and anti-hyperlipidemic activities [1].

Our current study aims at improving the nutraceutical and nutritional quality of whole extracts of AJN by *Lactobacillus plantarum* fermentation. Lactic acid bacteria are the best-known microorganisms associated with food fermentation, and their capability to metabolize a range of plant-derived compounds has been investigated [2, 3]. A recent study applied microbial fermentation for cherry juice by *Lactobacilli*, which resulted in potential benefits including decreased sugar content, improved flavor, and increased bioactivities.

To comprehensively profile the fermentation-driven metabolic features of AJN, integrative mass-spectrometric analysis was conducted based on targeted and untargeted profiling approaches. We analyzed 20-hydroxyecdysone and 25S-inokosterone, two representative bioactive compounds, using liquid-chromatography orbitrap mass-spectrometry (LC-orbitrap MS). In addition, a range of primary metabolic profiles were acquired using gas-chromatography time-of-flight mass spectrometry (GC-TOF MS). The results demonstrated the substantial modulation in the metabolite contents of AJN following the fermentation process including the major bioactive markers.

We first examined if the fermentation affected the level of 20-hydroxyecdysone, major indicator and functional compound of AJN [1, 4, 5]. Reverse phase liquid chromato-graphy coupled to mass-spectrometric analysis was conducted with positive ionization mode.

Two extracted ion chromatograms were detected at m/z of 481.3165 (retention time: 1.41 min and 2.67 min) with mass accuracy of 5 ppm. The m/z value corresponded to [M+H]^+^ ion of 20-hydroxyecdysone and 25S-inokosterone, and the identities were differentiated by tandem mass spectra and retention time [1, 6] (Fig. 1A). Quantitative comparison (peak area) of the compounds showed the significant increase following the post-fermentation process (fold change = 2.6 and 2.3, respectively for 20-hydroxyecdysone and 25S-inokosterone, *p* < 0.001) (Fig. 1B).

GC-TOF MS analysis was performed on extract derivatives to identify additional changes in the AJN metabolome by the *Lactobacillus plantarum* fermentation. The untargeted metabolic profiling resulted in 80 compounds that were uniquely identified and semi-quantified based on *Binbase* algorithm [7]. The metabolites were carbohydrates (34%), amino acids (28%), organic acids (10%), fatty acids (8%), and others (20%).

First, principal component analysis (PCA) revealed clear distinction in the metabolite profiles between pre- and post-fermentation of the AJN. T1 separated the two groups (64.6%) while T2 mostly explained the variance among biological replicates (different batches) within each group (80.4%) (Fig. 2A). Likewise, the subsequent model using PLS-DA presented the clear discrimination with best explained variance (R^2^Y= 1) and predictability (Q^2^= 0.993)(Fig. 2B). The VIP analysis showed that TCA cycle intermediates, malic acid and fumaric acid most contributed to the discriminant model. Others were organic acids (glucuronic acid and glyceric acid), nitrogenous compounds (valine, 3-hydroxypyridine, xanthine, and thymine), cellobiose, and lyxitol.

Next, the compositional characteristics after the fermen-tation process were further investigated using univariate statistics (Student’s *t*-test). A total of 46 metabolites were significantly different in the fermented extract in which 36 and 10 compounds were up- and down-regulated (Table 1). The highest fold-increase was found in cellobiose (55.7 fold-change, *p* < 0.001) whereas the most dynamic down-regulation was detected in lyxitol (0.1 fold-change, *p* < 0.001). Pathway over-representation analysis proposed that amino acid metabolisms were most significantly altered by the fermentation, which included Ala-Asp-Glu metabolism, Val-Leu-Ile metabolism, Arg-Pro metabolism, and Tyr metabolism (Fig. 2C). Other metabolisms were pantothenate-CoA biosynthesis, carbon fixation, and butanoate meta-bolism. In addition, we explored the putative biochemical mechanism on the increased levels of 20-hydroxyecdysone. We retrospectively investigated potential precursors or intermediates that may be converted to 20-hydroxyecdysone by the *Lactobacillus plantarum* fermentation. Cholesterol and sitosterol are the precursors for the compounds [8]. Indeed, we identified a precursor, sitosterol, that was marginally increased in the fermented AJN whereas cholesterol was not measurable.

In conclusion, this study demonstrated that the primary bioactive components, 20-hydroxyecdysone and 25S-inokosterone in AJN, were significantly increased by *Lactobacillus plantarum* fermentation. Moreover, various types of primary metabolites (*e.g.* amino acids) and phytosterols (*e.g.* sitosterol) were identified and may require further comprehensive investigation on mechanistic understanding, which may lead to the development of a microbial production system for the highly-bioactive compound.

## Figures and Tables

**Fig. 1:**
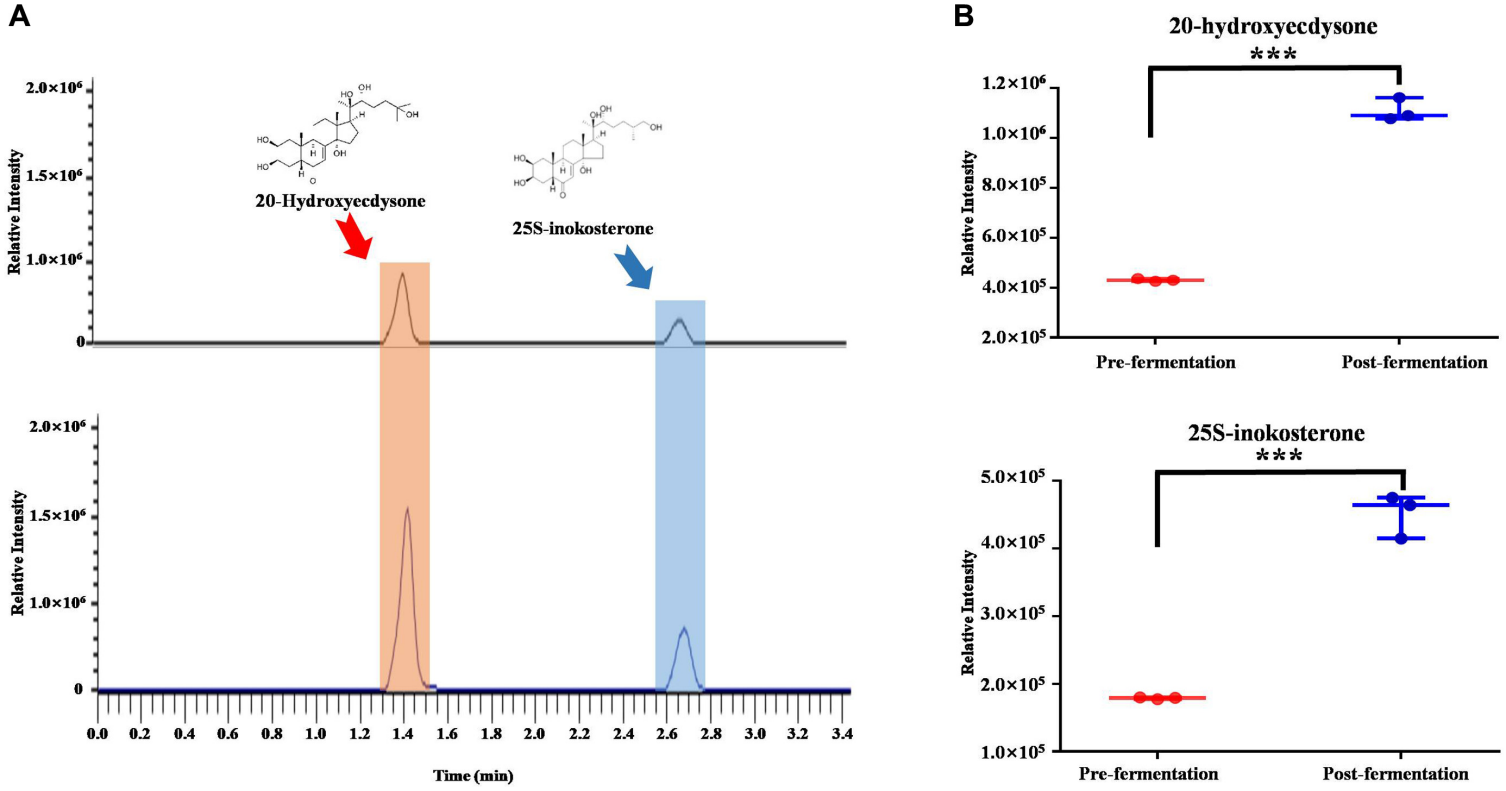
The significant increases of primary indicator and representative functional compounds, 20-hydroxyecdysone and 25Sinokosterone. (**A**) Base peak chromatograms and (**B**) semi-quantitative comparison of the compounds between pre- and post-fermentation. Statistical analysis was done based on Student’s *t*-test. ***p < 0.001. Y-axis is relative intensity.

**Fig. 2:**
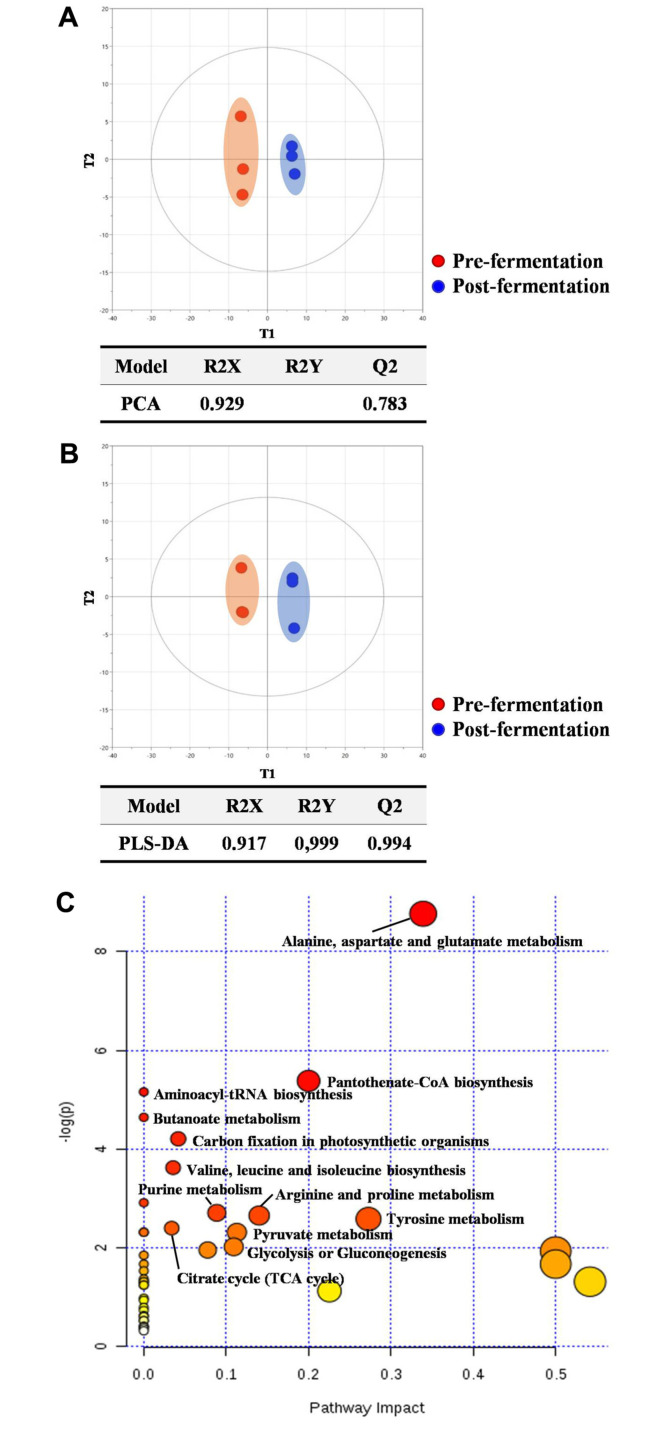
Comparative analysis of primary metabolic profiles between pre- and post-fermentation. The score scatter plots by PCA (**A**) and PLS-DA (**B**). Both unsupervised and supervised multivariate statistical analysis showed clear discrimination of the metabolite profiles between pre- and postfermentation. Pathway over-representation analysis of the metabolites that were significantly increased after the fermentation (**C**) X-axis proposed the topological significance of the metabolites in a specific pathway (relative-betweenness centrality) whereas y-axis presented the significant levels of pathway alteration (hypergeometric test).

**Table 1:** The list of metabolites that were significantly increased by *L. plantarum* fermentation (Student’s *t*-test, *p* < 0.05).

Metabolite	*p*- value	Fold change
Cellobiose	< 0.001	55.7
Gluconic acid	< 0.001	8.2
Pinitol	< 0.001	7.0
Glutamate	0.001	5.0
Glyceric acid	< 0.001	4.2
3-Hydroxypyridine	< 0.001	3.4
Isoleucine	< 0.001	3.3
Fructose-6-phosphate	< 0.001	3.3
Pyruvic acid	< 0.001	3.2
Valine	< 0.001	2.8
Fumaric acid	< 0.001	2.5
Tyrosine	< 0.001	2.4
2-Deoxytetronic acid	0.006	2.2
Tartaric acid	< 0.001	2.2
Phenylalanine	< 0.001	2.1
2,3-Dihydroxypyridine	0.011	1.9
Gamma-aminobutyric acid	0.001	1.8
Isothreonic acid	0.017	1.8
Xylose	0.001	1.8
Threonic acid	< 0.001	1.7
Hydroxylamine	0.028	1.6
Butyrolactam	0.002	1.6
Oxalic acid	0.022	1.6
Uric acid	< 0.001	1.6
Alanine	0.007	1.5
Xanthine	< 0.001	1.5
3,6-Anhydro-d-galactose	0.002	1.5
Beta alanine	0.038	1.5
Nicotinic acid	0.001	1.4
Thymine	< 0.001	1.4
N-methylalanine	< 0.001	1.4
Guanine	0.004	1.4
Allantoic acid (dehydrated)	0.001	1.3
Oxoproline	0.006	1.3
Palmitic acid	0.045	1.3
Lactic acid	0.034	1.1
Uracil	0.004	0.9
Succinic acid	0.001	0.8
Galactinol	< 0.001	0.7
Inositol-4-monophosphate	0.047	0.6
6-Chlorohexanol	0.025	0.6
Tryptophan	0.003	0.6
Glutamine	< 0.001	0.3
Malic acid	< 0.001	0.2
Erythritol	< 0.001	0.2
Lyxitol	< 0.001	0.1

Fold change was the ratio of post-fermentation over pre-fermentation.
